# Genomic Instability and Adaptive Evolution Induced by RFA Insufficiency in *Saccharomyces cerevisiae*

**DOI:** 10.3390/cimb48020158

**Published:** 2026-01-30

**Authors:** Runbiao Zhang, Liyan Tian, Min He, Kejing Li

**Affiliations:** Ocean College, Zhejiang University, Zhoushan 316021, China; 22334173@zju.edu.cn (R.Z.); 12534034@zju.edu.cn (L.T.); 12434005@zju.edu.cn (M.H.)

**Keywords:** genomic stability, Replication Factor A, mismatch repair, DNA replication, replication stress

## Abstract

This study systematically investigated the genomic alterations in *Saccharomyces cerevisiae* driven by Replication Factor A (RFA) dosage insufficiency using a promoter-replacement strategy combined with mutation accumulation and whole-genome sequencing. Our findings reveal that transcriptional suppression of *RFA2* or *RFA3* leads to severe growth inhibition. RFA deficiency induces a distinct mutational spectrum characterized by a high frequency of monosomy and terminal deletions, indicative of severe replication stress. Furthermore, loss of heterozygosity is significantly enriched at centromeres and high-GC regions, underscoring the role of RFA in stabilizing intrinsic genomic barriers. Utilizing an APOBEC3B-induced mutagenesis assay, we demonstrate that RFA insufficiency leads to the extensive accumulation of exposed ssDNA with a distinct bias towards the lagging strand template. Notably, we observed that cells spontaneously inactivate Mismatch Repair (MMR) genes, such as *MSH2* and *PMS1*, to survive RFA-induced stress. This hypermutant phenotype grants a certain degree of growth recovery on Low Galactose (LG) medium. Overall, these findings demonstrate that RFA dosage is a key determinant of genomic integrity and elucidate how repair pathway modulation drives adaptive evolution under replication stress.

## 1. Introduction

Genomic stability is fundamental to cell survival and genetic fidelity [1]. Cellular DNA is continuously threatened by endogenous and exogenous damaging agents [2]. Unrepaired damage leads to genomic instability events [3]. To counter the threats, eukaryotic cells have evolved a sophisticated DNA damage repair (DDR) system. This system activates cell cycle checkpoints to arrest division and coordinates DNA repair [4]. Replication Factor A plays an indispensable role in the DDR system. RFA is an evolutionarily highly conserved heterotrimeric (RFA1, RFA2, RFA3) complex. The largest subunit, RFA1, utilizes its N-terminal domain (BD-F) to recruit checkpoint proteins, such as Mec1-Ddc2, to DNA lesions [5]. RFA2 contains a C-terminal winged-helix domain [6] for protein interactions and a serine and threonine-rich N-terminus that undergoes phosphorylation to regulate checkpoint progression [7]. The smallest subunit, RFA3, forms the essential trimerization core with RFA1 and RFA2 to maintain complex stability [8]. Its core function is binding single-stranded DNA (ssDNA) with high affinity. ssDNA is a common intermediate in DNA replication and almost all DNA repair pathways [9]. Therefore, RFA acts as a ubiquitous DNA damage sensor and a critical factor in maintaining genomic stability [10].

The function of RFA in genomic stability is twofold. First, acting as a DDR signal initiator, RFA-coated ssDNA serves as a platform for recruiting DDR kinases, such as Mec1-Ddc2 [11,12]. This activates the downstream Rad53 checkpoint pathway. Consequently, the cell cycle arrests to allow time for repair [5]. Second, RFA protects ssDNA from degradation upon binding [10]. It also plays a crucial role in homologous recombination (HR) repair [13]. RFA must be orderly replaced by mediator proteins, such as Rad52 [14]. This step enables the loading of Rad51 recombinase and ensures high-fidelity repair [15]. In addition to the prevention of R-loop-Associated Damage and Fork Collapse, recent studies have also highlighted the indispensable role of RFA in suppressing the formation of R-loops at transcription-replication collision sites [16].

Previous studies have predominantly relied on point mutations [17,18] or alleles (like rfa1-t11) [19], which focus on specific functional defects. To isolate the systemic consequences of RFA insufficiency, we replaced the native promoters of *RFA1*, *RFA2*, and *RFA3* with the galactose-inducible GAL1 promoter. Unlike traditional mutational approaches that permanently alter protein structure, this strategy allows for modulation of expression levels. Given that RFA is a key coordinator of the high-fidelity HR pathway [20], its dosage insufficiency significantly impairs HR efficiency. Theoretically, this forces cells to rely on alternative repair pathways to cope with endogenous DNA damage [21]. These pathways might include non-homologous end joining (NHEJ) [22], single-strand annealing (SSA) [23], or translesion synthesis (TLS) [24]. The selection of these error-prone pathways might preserve survival. However, their error-prone nature could paradoxically become a major source of genomic instability in RFA-deficient cells.

In this study, we used *Saccharomyces cerevisiae* as a model to investigate the specific impacts of RFA gene defects on genomic stability. We focused on phenotypic changes following the suppression of RFA subunit expression. We analyzed genomic variation events using high-throughput whole-genome sequencing (WGS) after multiple passages. We examined Single Nucleotide Variations (SNVs), Small Insertions/Deletions (InDels), Loss of Heterozygosity (LOH), Chromosomal Rearrangements (CRs), and Aneuploidy [25,26]. We aimed to explore potential alternative repair pathways or compensatory mechanisms under RFA-deficient stress. Our findings provide new insights into the core role of RFA in coordinating DNA repair pathway choice.

## 2. Materials and Methods

### 2.1. Strains and Medium

*Saccharomyces cerevisiae* strains were derived from W303-1A and YJM789 backgrounds [25]. These were obtained from laboratory stocks. Diploid strains were generated by crossing haploids from these two backgrounds. Plasmids pUG72 (URA3) and pUG6 (KanMX6) served as PCR templates. Plasmids pSR440 contain APOBEC3B. Strains were routinely cultured in YPD medium (1% yeast extract, 2% peptone, 2% glucose). Antibiotics were added for selection as required. High Galactose (HG) medium was used to support normal expression. This medium contained 1% yeast extract, 2% peptone, 3% raffinose, and 0.05% galactose. Low Galactose (LG) medium (0.005% galactose) was used to suppress expression. All strains were incubated at 30 °C.

### 2.2. Genetic Manipulations and Strain Construction

Primers used in this study are listed in Table S1. We employed a promoter replacement strategy. The KanMX6-GAL1-3xHA cassette was amplified from laboratory plasmids [26]. This cassette was integrated upstream of the respective RFA open reading frames via homologous recombination. Transformation was performed using the lithium acetate/polyethylene glycol method [27]. Transformants were selected on HG plates containing 600 ng/mL Geneticin (G418). Positive clones were verified by PCR and Sanger sequencing (Shangya Biotech, Hangzhou, China). Verified haploids were crossed to generate diploid strains L*RFA1* (WY*p*GAL1-*RFA1*), L*RFA2* (WY*p*GAL1-*RFA2*), and L*RFA3* (WY*p*GAL1-*RFA3*). The strains constructed in this study are shown in the Table S2.

### 2.3. Phenotypic Assays and Western Blotting

For growth assays, cells were grown to the logarithmic phase in HG medium. The sample was washed twice with sterile water and adjusted to an OD_600_ value of 0.1. After three ten-fold serial dilutions, 2 µL aliquots of each dilution were spotted onto HG, LG, and YPD plates. Plates were incubated at 30 °C for 72 h. We verified protein downregulation using strains WT-*RFA2*. In these strains (W*RFA2*-ha), the KanMX6-GAL1 promoter was reverted to the native ATG promoter, but the 3xHA tag was retained. Cells were cultured in HG or LG liquid media at 30 °C with shaking (200 rpm) for 18 h. Total proteins were extracted using a mechanical disruption method with glass beads. Briefly, yeast cells were harvested by centrifugation, and approximately 25 mg of cell pellets were resuspended in 50 μL of lysis buffer (10 mM Tris-HCl pH 7.8, 0.1 M NaCl, 0.1 M Glycine, 0.1% Triton X-100, 10 μM DTT, and 1 mM PMSF). An appropriate amount of 0.5 mm glass beads was added to the suspension. The mixture was vortexed vigorously for 10–20 min to ensure complete cell lysis. Subsequently, the samples were centrifuged at 12,000 rpm for 20 min at 4 °C. The supernatant containing total proteins was carefully transferred to a fresh tube prior to SDS-PAGE analysis. Protein levels were analyzed by SDS-PAGE and Western blotting using anti-HA antibodies. GAPDH served as a loading control.

### 2.4. Mutation Accumulation (MA) Lines

**Figure 1:**
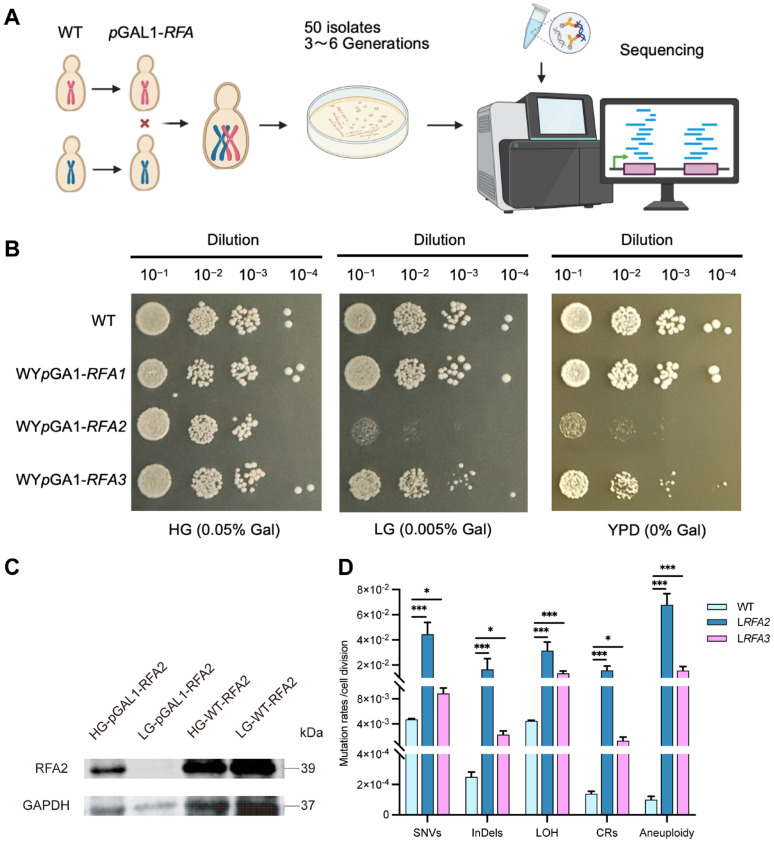
Growth phenotypes of low expressing RFA strains and verification of RFA2 protein expression levels. (**A**) Experiment flowchart. We constructed conditional knockdown strains by replacing the native promoters of the RFA genes with the GAL1 promoter in W303 and YJM789 backgrounds, respectively. These haploids were then crossed to generate diploid strains. Subsequently, MA experiments were conducted on solid LG medium. A total of approximately 50 isolates were selected and cultured for 3–6 passages prior to whole-genome sequencing. (**B**) WT represents the wild-type diploid strain without promoter replacement. (**C**) HG-pGAL1-RFA2 and LG-pGAL1-RFA2 represent protein samples collected from the W*p*GAL1-*RFA2* strain following overnight culture in HG and LG media. (**D**) Mutation rates of 5 classes of DNA variation events in strains WY*p*GAL1-*RFA2* and WY*p*GAL1-*RFA3* under low galactose conditions. Data are presented as mean ± SEM. *p* values were determined using the Wilcoxon rank-sum test and are indicated by asterisks (* *p* < 0.05, *** *p* < 0.001).

Mutation accumulation experiments were conducted to assess genomic stability. Strains L*RFA2* and L*RFA3* were passaged on LG solid medium to induce RFA suppression. Lines were transferred every 120 h. Due to the severe growth inhibition of RFA-deficient strains on LG medium (as shown in Figure 1B), this extended interval was necessary to allow colonies to reach a sufficient size (equivalent to ~20–25 generations) for transfer. This process continued for 5 passages. Parallel control lines were maintained on HG medium. After the final passage, single colonies were isolated and expanded in HG medium. Genomic DNA was extracted for sequencing.

### 2.5. DNA Extraction and Genome Sequencing

Genomic DNA was isolated from yeast cultures grown overnight in 5 mL YPD broth using the Genomic DNA Extraction Kit (Omega Bio-tek Inc., Norcross, GA, USA). DNA integrity and concentration were verified via agarose gel electrophoresis and the Qubit™ dsDNA BR Assay Kit (Thermo Fisher Scientific, Waltham, MA, USA). For library construction, genomic DNA was ligated with index adapters (MGI Adapter Set 8, Vazyme, Nanjing, China). Libraries were prepared automatically on an MGISP-960 system (MGI, Shenzhen, China) utilizing the VAHTS^®^ Universal Plus DNA Library Prep Kit for MGI (Vazyme, Nanjing, China). Post-preparation quality control and quantification were conducted with the Qubit^TM^ dsDNA HS Assay Kit (Thermo Fisher Scientific, Waltham, MA, USA). Libraries were circularized using the VAHTS^®^ Circularization Kit for MGI (Vazyme, Nanjing, China). DNA nanoballs (DNBs) were subsequently generated using the MGISEQ-2000RS High Throughput Sequencing Kit (MGI, Shenzhen, China) and sequenced on the MGISEQ-2000 platform (MGI, Shenzhen, China) with a paired-end 150 bp (PE150) configuration. Quality control of raw reads was performed using FastQC (v0.11.9). Trimmomatic (v0.39) was then used to filter low-quality bases (Phred score < 20) and remove adapter sequences. Subsequently, Flye software (v2.39) was utilized to assemble the data from the Nanopore sequencing platform to obtain the contig sequences. The high-quality second-generation data were then used to correct the third-generation contig results using Pilon [28].

### 2.6. Whole Genome Sequencing Analysis

1. High-quality sequencing reads (~2G per sample) were mapped to the *Saccharomyces cerevisiae* S288C reference genome using the BWA-MEM algorithm with standard settings [29]. 2. SAMtools was used to convert SAM files to BAM format and sort them [30], then determine sequencing coverage and detect mutations. 3. VarScan was subsequently employed to identify SNVs and InDels [31]. 4. Based on established protocols [25,32], variants present in both the wild-type and mutant strains were classified as background mutations and excluded. Variant annotation was performed with SnpEff [33] to predict functional impacts on coding sequences. Mutation rates per base pair per cell division were calculated using the following formula:

$$\mu =\frac{N}{\left(s\;*\;n\;*\;24\;*\;23000000\right)}$$

*N* represents the total number of detected SNVs or InDels, *s* is the number of isolates, *n* is the number of subculture passages, 24 denotes the estimated generations per passage, and the number of base pairs of the genome of *S. cerevisiae* is 23 Mb.

### 2.7. Association Analysis of LOH and Chromosomal Elements

We analyzed the correlation between LOH events and various chromosomal elements. The expected number of elements within LOH windows was calculated based on the genome-wide distribution. Specifically, the ratio of the LOH window length to the total genome length was multiplied by the total number of elements in the sequenced region. Observed numbers were counted directly from the sequencing data. Statistical significance of enrichment or depletion was determined using a Chi-square test (Excel). * *p*-value < 0.05 was considered significant. The numbers and position of the elements refer to the previous data [26,32].

### 2.8. Detection of Rad52 Foci

To detect Rad52 foci, we used the wild-type and RFA2 mutant strain X8068-6Crfa2 (Table S2) expressing Rad52-YFP. Yeast cells were cultured in 5 mL liquid medium to log phase (OD600 of 0.1) and immediately visualized under a high-resolution confocal microscope (OLYMPUS FV3000, Tokyo, Japan) equipped with a 60× objective (NA 1.42). Rad52-YFP foci in Z-image stacks were acquired and analyzed using ImageJ/Fiji (v1.54f).

## 3. Results

### 3.1. Genomic Instability Induced by RFA Insufficiency

To systematically investigate the genomic alterations driven by RFA dosage insufficiency, we established a quantitative MA system coupled with whole-genome sequencing. We constructed conditional knockdown strains by replacing the native promoters of *RFA1*, *RFA2*, and *RFA3* with the galactose-inducible GAL1 promoter (Figure 1A). We assessed growth phenotypes on solid media with varying galactose concentrations (Figure 1B). On HG medium, all promoter-replacement strains exhibited growth comparable to the wild type (WT). This observation confirms that the promoter replacement strategy itself does not compromise cell viability under high galactose. However, on LG medium, WY*p*GAL1-*RFA2* showed severe growth inhibition, while WY*p*GAL1-*RFA3* displayed partial inhibition. Interestingly, WY*p*GAL1-*RFA1* strain did not exhibit significant growth defects under these conditions. This might imply that the RFA1 subunit, as the largest subunit in the RFA complex, is particularly sensitive to cells. When its expression level is regulated by galactose, cells fail to construct the promoter replacement strain by mutating the GAL1 pathway. Protein expression levels were verified by Western blotting using a 3xHA tag fused to RFA2 (Figure 1C). In the W*p*GAL1-*RFA2* strain cultured in HG medium, RFA2 protein levels were comparable to those in the WT. Conversely, in LG medium, RFA2 expression was barely detectable. This result confirms that under low galactose conditions, the transcription and translation of *RFA2*—regulated by the GAL1 promoter, were effectively restricted. Consequently, the severe growth defects observed in Figure 1B can be directly attributed to the successful downregulation of the RFA2 protein.

Upon completion of the passages, WGS was conducted on 31 isolates of the RFA2-deficient strain (L*RFA2*) and 48 isolates of the RFA3-deficient strain (L*RFA3*), alongside the wild type. The sequencing analysis revealed that RFA deficiency triggered a catastrophic increase in genomic instability (Dataset S1, Dataset S2). Compared to the WT, both RFA-deficient strains exhibited a significant increase in the frequency of all detected variation types, including SNVs, InDels, LOH, CRs, and aneuploidy (Figure 1D). Specifically, the CRs rates in L*RFA2* and L*RFA3* were 102-fold and 11-fold higher than WT, corresponding to absolute rates of 1.41 × 10^−2^ and 1.54 × 10^−3^ mutations per cell division. Notably, aneuploidy rates increased dramatically, reaching 800-fold and 188-fold higher levels, respectively. Other genomic alterations were also substantially elevated; for instance, SNVs, InDels, and LOH in L*RFA2* increased by 9-fold, 60.6-fold, and 7-fold, respectively, compared to the WT. These elevations correspond to absolute mutation rates of 4.28 × 10^−2^, 1.45 × 10^−2^, and 3.13 × 10^−2^ events per cell division, respectively.

### 3.2. Distinct Mutational Spectra Induced by RFA Insufficiency

**Figure 2:**
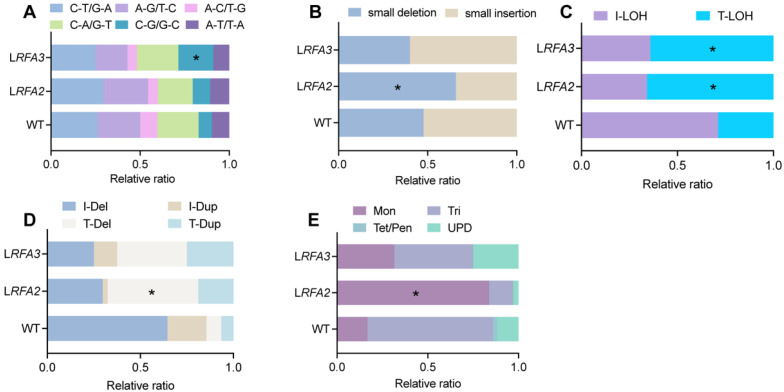
Mutational spectra under low RFA2 and RFA3. (**A**) Relative ratio of six types of SNVs. *p* values were determined using the fisher’s exact test and are indicated by asterisks (* *p* < 0.05) (**B**) Relative ratio of small insertions and deletions among InDels. (**C**) Relative ratio of T-LOH or interstitial (I-LOH) loss of LOH. (**D**) Relative ratio of terminal versus interstitial large-fragment insertions or deletions within CRs. (**E**) Relative ratio of aneuploidy events classified as Monosomy (Mon), Uniparental Diploid (UPD), Trisomy (Tri), or higher ploidy states (Tet/Pen).

Further analysis of variation subtypes revealed distinct mutational signatures associated with RFA stress (Figure 2). In the InDels, L*RFA2* strains showed a significantly higher proportion of small deletions (Figure 2B). These deletion events constituted 65.7% of total InDels, a proportion significantly higher than that observed in the WT (*p* < 0.05). Regarding structural variations, both L*RFA2* and L*RFA3* strains showed a preferential accumulation of instability at chromosome ends. Terminal deletions (T-Del) constituted 48.6% and 37.5% of all chromosomal rearrangements in L*RFA2* and L*RFA3*, respectively (Figure 2D). A similar trend was observed for LOH events, where Terminal LOH (T-LOH) accounted for 65.8% and 64.1% of the total LOH events in these strains (Figure 2C). Most strikingly, the analysis of aneuploidy revealed a specific vulnerability to chromosome loss; monosomy was the predominant error, accounting for 83.7% of all aneuploidy events in L*RFA2* and 31.5% in L*RFA3* (Figure 2E).

### 3.3. RFA Deficiency Induces DNA Damage and Genomic Instability at Replication Barriers

**Figure 3:**
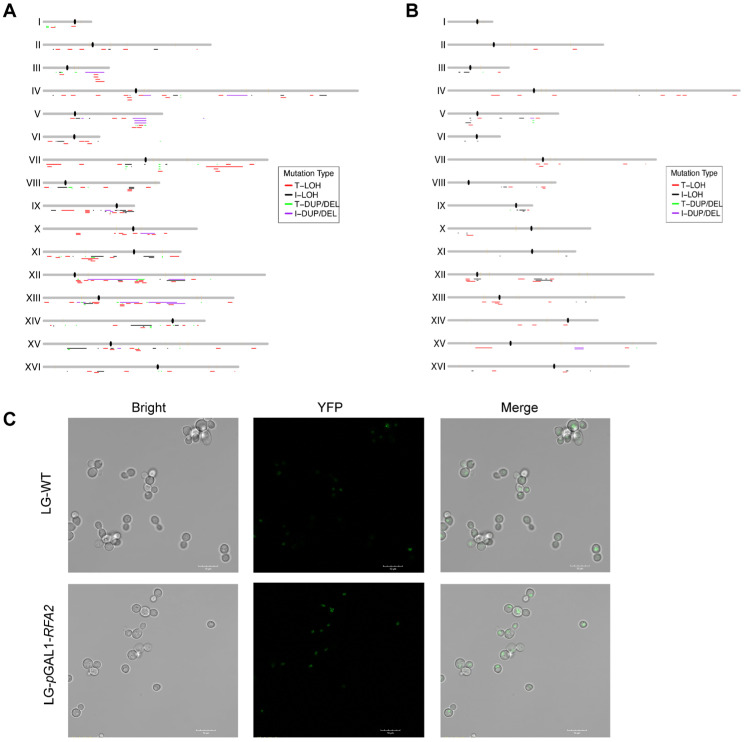
Genome-wide distribution of chromosomal alterations and visualization of DNA damage. (**A**) The distribution of chromosomal events in *RFA2*-deficient strain. (**B**) The distribution of chromosomal events in *RFA3*-deficient strain. (**C**) Representative fluorescence microscopy images of YFP-Rad52 foci in cells cultured in LG medium. The increased frequency of bright foci in the promoter-replaced strain compared to the WT indicates the accumulation of spontaneous DNA double-strand breaks (DSBs) and stalled replication forks due to RFA2 insufficiency.

We mapped the distribution of LOH and CR events across the genome for L*RFA2* and L*RFA3* strains (Figure 3). To identify regions prone to instability, we analyzed the correlation between LOH breakpoints and specific chromosomal elements. In the RFA2-deficient strain, LOH events were significantly enriched in regions with high GC content (*p* < 0.001, ratio = 1.41), while being significantly depleted in low GC regions (Dataset S1-3). In the *RFA3*-deficient strain, a broader and more distinct landscape of associations was observed (Dataset S2 and S3). LOH breakpoints showed a striking colocalization with known replication barriers and structural landmarks. Specifically, we detected significant enrichment at centromeres (*p* < 0.001, ratio = 5.00), G-quadruplex (G4) motifs (*p* < 0.001, ratio = 1.64), and Rrm3p binding sites (*p* < 0.05, ratio = 1.69), which are known pause sites for replication forks (Dataset S2 and S3). Furthermore, consistent with the L*RFA2* data, L*RFA3* also exhibited a strong bias towards high GC content regions (*p* < 0.001, ratio = 2.25). These distributions suggest that RFA deficiency renders the genome particularly fragile at some special sites like complex replication or structural positions.

The enrichment of LOH at these replication barriers suggests that RFA insufficiency impairs the stability of stalled replication forks, leading to fork collapse. To visualize spontaneous DNA damage and repair centers, we utilized a YFP-tagged Rad52 strain. The formation of Rad52 foci is a well-established marker for ongoing homologous recombination repair at sites of DNA double-strand breaks or stalled replication forks [34]. We introduced this reporter into the *RFA2* promoter-replacement strain and assessed the cellular response under RFA2-limiting conditions (Figure 3C). Following cultivation in LG medium, fluorescence microscopy revealed a marked increase in the frequency of YFP-Rad52 foci in *RFA2*-deficient cells compared to the control strain. This elevated level of physical DNA damage provides a mechanistic explanation for the observed increase in LOH and structural variations, confirming that insufficient RFA2 dosage fails to protect forks at difficult-to-replicate regions, thereby driving genomic instability.

### 3.4. RFA Insufficiency Leads to Accumulation of Unprotected ssDNA on the Lagging Strand

**Figure 4:**
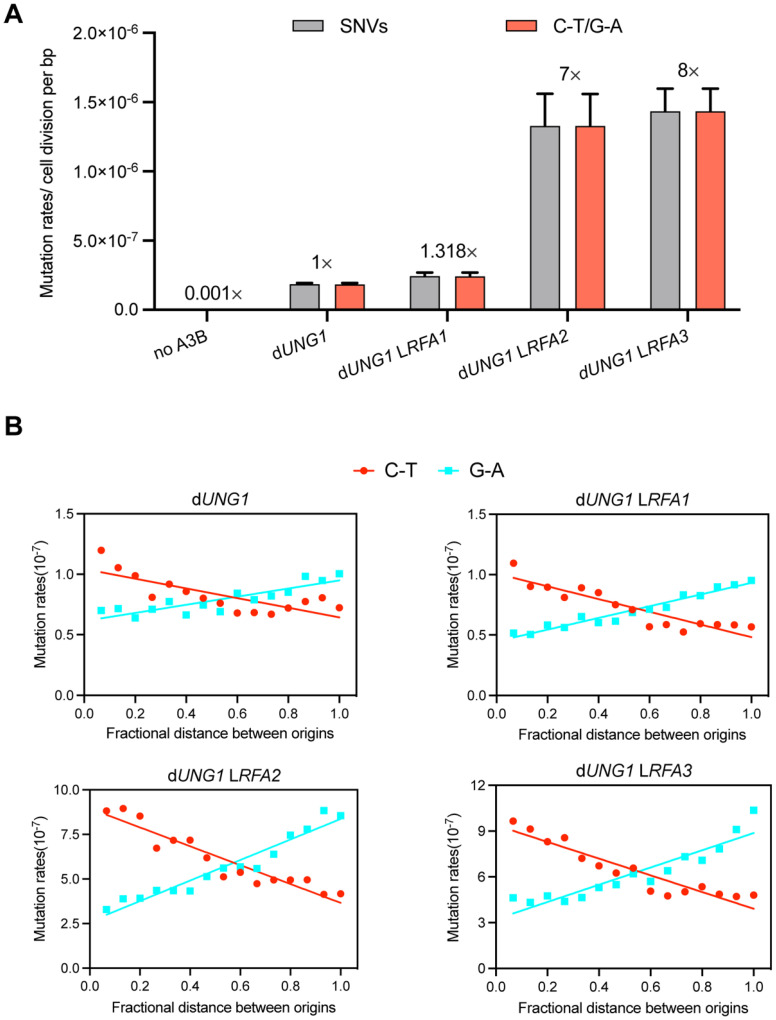
RFA deficiency exposes lagging-strand ssDNA to A3B-induced mutagenesis. (**A**) Mutation rates of SNVs induced by A3B. Strains lacking *UNG1* (d*UNG1*) and expressing A3B were used as the control (normalized to 1×). The WY*p*GAL1-*RFA* strains (L*RFA1*, L*RFA2*, L*RFA3*) in the d*UNG1* background show significantly elevated mutation rates. The vast majority of mutations were C-T/G-A transitions, characteristic of A3B activity. (**B**) Strand bias of A3B-induced mutations relative to replication origins. The *x*-axis represents the fractional distance between adjacent replication origins. The separation of C-T (red line) and G-A (blue line) frequencies demonstrates that ssDNA exposure is asymmetric and predominantly enriched on the lagging strand during replication.

To investigate whether RFA deficiency leads to an increase in exposed ssDNA in vivo, we employed an APOBEC3B-induced mutagenesis assay, a method previously established to quantify ssDNA formed during replication stress [35]. The human enzyme APOBEC3B (A3B) specifically deaminates cytosines to uracils in ssDNA, resulting in C-T transitions (or G-A on the complementary strand) during subsequent replication cycles [36]. We introduced a plasmid called pSR440 which express A3B into d*UNG1* derivates of our RFA promoter-replacement strains. The deletion of *UNG1* prevents the base excision repair of uracils, thereby preserving the A3B-induced mutation signature. As expected, expression of A3B in the control d*UNG1* strain increased the frequency of SNVs by approximately 1000-fold compared to the spontaneous mutation rate [35]. However, reducing RFA dosage drastically exacerbated this effect (Dataset S3). The *RFA2*-deficient and *RFA3*-deficient strains exhibited an 8-fold and 9-fold increase in SNVs rates, respectively, compared to the d*UNG1* control (Figure 4A). The *RFA1*-deficient strain showed a modest increase (1.3-fold). The mutational spectrum was dominated by C-T/G-A transitions, confirming that the elevated mutation rate was driven by A3B on exposed ssDNA.

To determine the genomic origin of this exposed ssDNA, we analyzed the strand bias of mutations relative to DNA replication origins. Following the methodology of Sui et al. [26], we mapped the distribution of C-T and G-A mutations across the intervals between adjacent replication origins (Figure 4B). This analysis revealed a profound strand asymmetry in RFA-deficient strains. We observed a significant enrichment of mutations corresponding to the lagging strand template. This pattern indicates that RFA plays a critical role in protecting the ssDNA intermediates formed during lagging strand synthesis. These results provide direct evidence that insufficient RFA leads to extensive ssDNA exposure, leaving replication intermediates accessible to A3B attack and prone to genomic instability.

### 3.5. Genomic Variation Under Moderate RFA2 Suppression and the Emergence of MMR-Deficient Hyper-Mutators

**Figure 5:**
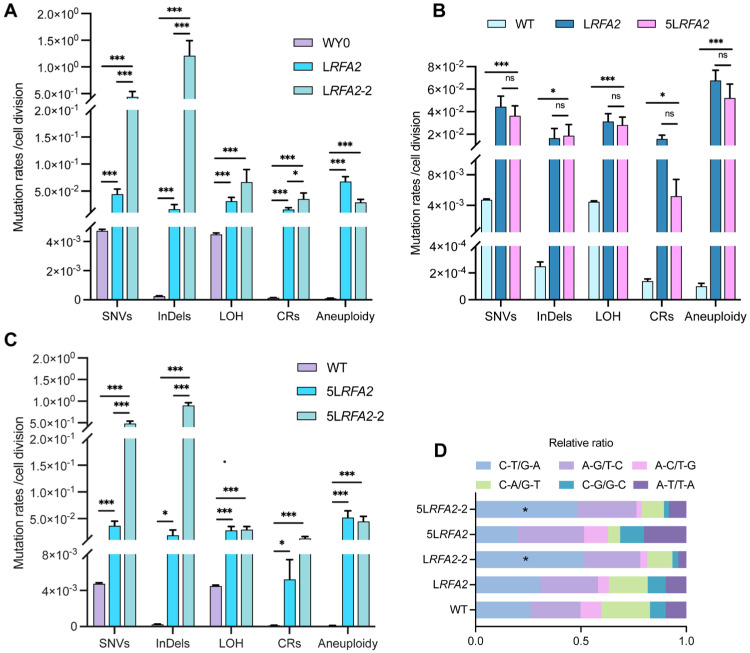
Comparative analysis of genomic instability between hyper-mutator and non-hyper-mutator lineages. Data are presented as mean ± SEM. *p* values were determined using the Wilcoxon rank-sum test and are indicated by asterisks (* *p* < 0.05, *** *p* < 0.001; ns: not significant). (**A**) Mutation rates of five classes of DNA variation events in strains L*RFA2* and L*RFA2*-2 under low galactose conditions. (**B**) Frequencies of five classes of DNA variation events in strains L*RFA2* and 5L*RFA2*. (**C**) Frequencies of five classes of DNA variation events in strains 5L*RFA2* and 5L*RFA2*-2. (**D**) Relative ratio of SNV types under two galactose concentrations.

During the comprehensive analysis of L*RFA2* isolates, we identified a distinct subpopulation consisting of four isolates (they were designated as the L*RFA2*-2 lineage) that exhibited exceptionally high mutation frequencies. Detailed analysis of the sequencing data (Dataset S1) revealed that these four isolates were derived from independent parallel MA lines. Furthermore, they exhibited non-overlapping mutational profiles with distinct driver mutations in MMR pathway genes, confirming that the hypermutator phenotype in each isolate originated from an independent adaptive event rather than clonal expansion from a common ancestor. Quantitative comparison with the main L*RFA2* lineage revealed a specific pattern of instability: the L*RFA2*-2 lineage showed drastically elevated rates of SNVs and InDels, whereas the frequencies of large-scale genomic alterations, including LOH, CRs, and aneuploidy, remained comparable to the primary lineage (Figure 5A). Functional annotation of SNVs and InDels in the L*RFA2*-2 lineage using SnpEff revealed numerous mutations in genes associated with DNA damage repair, particularly those related to the MMR pathway (Table S3).

Given that the L*RFA2* genome exhibited extreme instability characterized by extensive chromosomal loss and large segmental deletions under LG conditions, we utilized Medium Galactose (MG, 0.025% Gal) concentrations to create the 5L*RFA2* lineage. This approach was intended to decrease chromosomal loss while allowing the observation of intermediate evolutionary phenomenon. Consistent with findings under LG conditions, a substantial subset of isolates (11 out of 19, designated as L*RFA2*-2) spontaneously evolved a distinct hyper-mutator phenotype. We observed that both the 5L*RFA2* lineages maintained significantly elevated levels of genomic instability compared to the wild type. However, after appropriately increasing the concentration of galactose, 5L*RFA2* did not show significant differences in the mutation rates of the five DNA variation events compared with the LG group (Figure 5B). This confirms that even moderate depletion of RFA2 is sufficient to compromise global genomic integrity. Comparative analysis of the 5L*RFA2*-2 and 5L*RFA2* lineages revealed a mutational pattern mirroring that of the L*RFA2* group. Specifically, only SNVs and InDels exhibited a drastic and statistically significant increase, whereas other types of genomic variations remained comparable between the two groups (Figure 5C). Consistently, functional annotation of the 5L*RFA2*-2 isolates using SnpEff identified mutations in genes associated with the MMR pathway (Table S3). This dissociation indicates that the mechanism driving the hyper-mutator phenotype specifically affects replication fidelity rather than chromosomal segregation or recombination. Further characterization of the mutational spectrum supported this conclusion: the hyper-mutators were defined by a significant enrichment of C-T/G-A transitions (Figure 5D). This specific spectral signature is characteristic of uncorrected replication errors, providing strong evidence that the L*RFA2*-2 and 5L*RFA2*-2 lineage adapted via the functional inactivation of the MMR pathway [37,38].

### 3.6. Inactivation of MMR Genes Confer a Growth Advantage Under RFA2 Deficiency

To verify whether MMR deficiency contributes to the survival of *RFA2*-deficient cells, we individually deleted seven key MMR pathway genes in W123. Growth assays revealed that while the parental W123 strain failed to grow on LG medium, the double mutants lacking MMR genes exhibited visible growth (Figure 5A). Specifically, the deletion of *MSH2* or *PMS1* resulted in the most distinct recovery of growth (Figure 5B,C). This also indirectly confirms the core position of *MSH2* and *PMS1* in the MMR repair pathway in previous studies [39].

## 4. Discussion

### 4.1. RFA Plays an Important Role in the Genomic Stability of Saccharomyces cerevisiae

We evaluated the impact of downregulating individual RFA subunits on cell growth by replacing their native promoters with the galactose-inducible GAL1 promoter. We observed that transcriptional suppression of *RFA2* and *RFA3* resulted in severe growth inhibition on low-galactose media. The inhibition of RFA2 and RFA3 leads to extreme genomic instability (Figure 1D). The genomic landscape of RFA-deficient strains was characterized by a pervasive bias towards “genetic loss.” This signature manifested across two distinct biological scales: a significant increase in the proportion of small deletions and a surge in terminal deletions and monosomy.

At the nucleotide scale, the enrichment of small deletions suggests a shift in DSBs repair pathway choice. High-fidelity HR strictly requires RFA to coat resected ssDNA and facilitate the loading of RAD51 [40]. In our RFA-deficient model, the impairment of RAD51 loading renders HR inefficient. Consequently, cells are forced to rely on alternative, error-prone pathways such as Microhomology-Mediated End Joining (MMEJ) or SSA. Crucially, both MMEJ and SSA function by annealing short homologous sequences and enzymatically removing non-homologous flaps [41]. This mechanism results in the obligatory deletion of intervening sequences.

At the chromosomal scale, the prevalence of monosomy (accounting for 83.7% of all aneuploidy events in L*RFA2*) points to severe, unresolved replication stress. This phenotype aligns with findings by Zheng [32], who demonstrated that replication stress induced by polymerase deficiency leads to extensive genomic instability. We hypothesize that monosomy arises from Checkpoint Adaptation [42]. RFA depletion leads to the accumulation of extensive ssDNA gaps at stalled replication forks. These gaps activate the DNA damage checkpoint. However, without sufficient RFA to coordinate repair, the damage remains unresolvable. Yeast cells eventually override this arrest after prolonged stalling and force entry into mitosis. During this aberrant division, chromosomes with collapsed forks fail to segregate, leading to whole-chromosome loss. While generally detrimental, monosomy acts as a special survival strategy: eliminating a chromosome undergoing big failure is preferable to a permanent, lethal cell cycle arrest [43]. Crucially, the accumulation of ssDNA intermediates was directly substantiated by our APOBEC3B-induced mutagenesis assay. We observed a drastic elevation in A3B-induced mutations in RFA-deficient strains, confirming that replication intermediates are physically exposed to A3B. Furthermore, the significant enrichment of these mutations on the lagging-strand template implies that the discontinuous nature of lagging-strand synthesis makes it particularly sensitive to RFA depletion. Since the lagging strand inherently generates transient ssDNA tracts during Okazaki fragment maturation [44], insufficient RFA dosage leads to the widespread exposure of these intermediates. These unbound ssDNA tracts serve as substrates for enzymatic modification and secondary structure formation [45], thereby precipitating the cascade of genomic instability observed in our study.

### 4.2. RFA Protects Replication Forks at Structurally Complex Barriers

The non-random distribution of LOH events reveals that RFA is indispensable for preserving genome integrity at structurally complex sites. We observed significant enrichment of instability at centromeres, high GC-content regions, and G4 motifs. These findings suggest that RFA is essential for preserving genome integrity at structurally complex loci prone to DNA damage stress (Figure 3A,B). In the absence of sufficient RFA to unfold these structures or stabilize the unwound ssDNA, stalled forks become highly susceptible to collapse. This mechanistic link is directly supported by our observation of elevated YFP-Rad52 foci in *RFA2*-deficient cells (Figure 3C). Since Rad52 foci mark active repair centers at sites of DSBs [34], their accumulation confirms that RFA depletion converts stalled forks into catastrophic strand breaks. Specifically, the enrichment of LOH at centromeres suggests that RFA is critical for navigating the replication machinery through these tightly bound kinetochore complexes. Similarly, the association with high-GC and G4 regions highlights RFA’s function in preventing secondary structure formation [46]. In summary, RFA depletion transforms these intrinsic structural features from manageable elements into hotspots of genomic fragility.

### 4.3. MMR Inactivation: A Gateway to Adaptive Evolution

**Figure 6:**
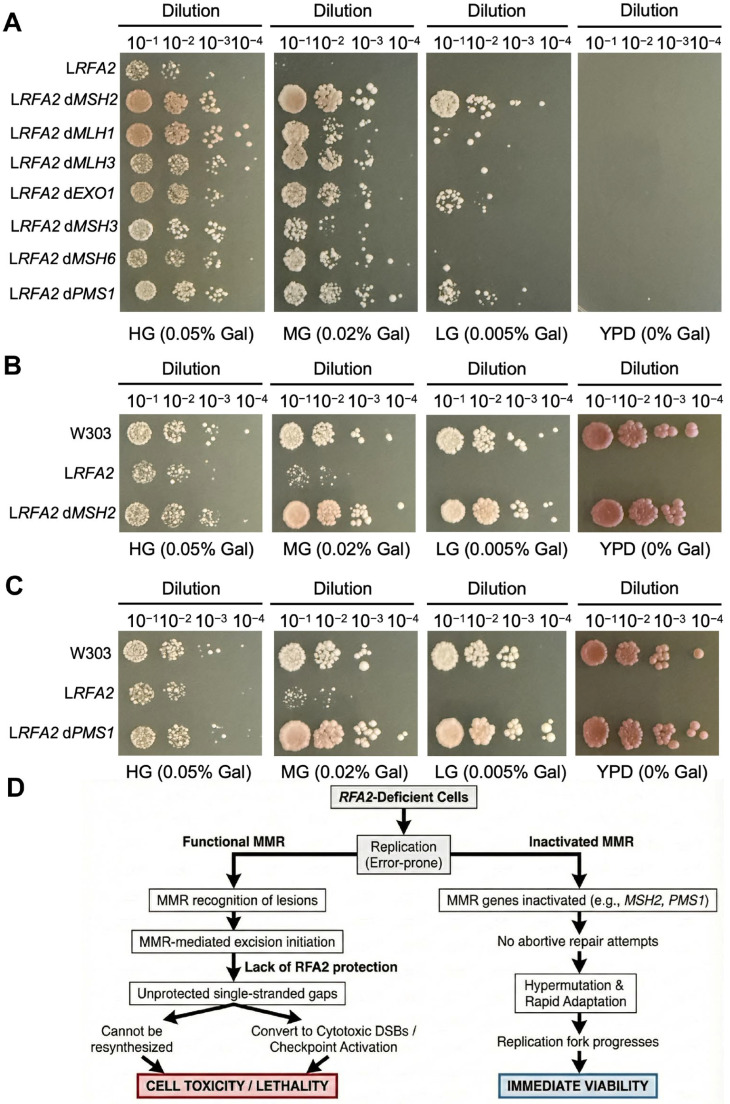
The role of MMR in low-expressing RFA2 strains. (**A**) Growth phenotypes of strain W*p*GAL1-*RFA2* and its deleting specific MMR genes (*MSH2*, *MLH1*, *MLH3*, *EXO1*, *MSH3*, *MSH6* and *PMS1*) under varying galactose concentrations. The symbol “d” denotes gene deletion. (**B**) Growth phenotypes of deleting *MSH2* strain in the W*p*GAL1-*RFA2* background. (**C**) Growth phenotypes of deleting *PMS1* strain in the W*p*GAL1-*RFA2* background. (**D**) The trade-off between genetic fidelity and survival in *RFA2*-deficient cells. The diagram illustrates that functional MMR is toxic in the absence of RFA2 because repair intermediates become unprotected single-stranded gaps (**left**). In contrast, inactivation of the MMR pathway facilitates survival by preventing abortive repair. This allows for hypermutation and rapid adaptation, which supports replication fork progression and results in immediate viability (**right**).

A pivotal turning point in our evolution experiment was the spontaneous emergence of hyper-mutator lineages (L*RFA2*-2, 5L*RFA2*-2) characterized by a drastic explosion of SNVs and InDels (Figure 5A,C). Genome annotation linked this phenotype to loss-of-function mutations in MMR genes. This event was highly selected, as evidenced by our finding that artificial deletion of *MSH2* or *PMS1* partially restored the growth of *RFA2*-deficient strains (Figure 6A).

We propose that in the absence of sufficient RFA2, the initiation of MMR-mediated excision generates lethal single-stranded gaps that cannot be resynthesized, thereby converting repair attempts into cytotoxic DSBs (Figure 6D). In RFA2-deficient cells, replication is inherently error-prone. Functional MMR machinery recognizes these lesions and initiates excision repair. However, the excision step of MMR critically requires RFA-complex to protect the single-stranded template and coordinate resynthesis [47]. In the absence of *RFA2*, MMR initiation creates lethal, unprotected gaps that cannot be refilled [48,49]. These gaps may convert into cytotoxic double-strand breaks or trigger permanent checkpoint activation [50]. Thus, active MMR becomes toxic to the cell. By inactivating MMR genes, cells effectively stop these abortive repair attempts. They trade genetic fidelity for immediate viability, allowing the replication fork to progress despite the high error rate [51]. This strategy is similar to the mechanisms observed in fungal pathogens and cancers, where hypermutations promote rapid adaptation [52].

## 5. Conclusions

This study elucidated the genomic alterations and adaptive evolution in *Saccharomyces cerevisiae* driven by RFA dosage insufficiency. We found that transcriptional suppression of *RFA2* or *RFA3* precipitated catastrophic genomic instability characterized by a distinct mutational spectrum of monosomy and terminal deletions. Genomic alterations were found to be significantly enriched at G4 motifs, chromosome terminals, and high-GC regions. In addition, *RFA2*-deficient cells exhibited a marked accumulation of Rad52 foci, indicative of extensive DSBs and stalled replication forks. This indicates the significant role of RFA in stabilizing the intrinsic replication barrier and advancing the DNA replication fork. Furthermore, utilizing an APOBEC3B-induced mutagenesis assay, we provided direct evidence that RFA insufficiency leads to a drastic increase in exposed ssDNA, and it shows the characteristic of ssDNA enrichment on the lagging strand. We identified spontaneous inactivation of the MMR pathway as a critical survival strategy. Experimental validation confirmed that the deletion of MMR genes, particularly *MSH2* and *PMS1*, partially alleviates the severe growth defects caused by RFA2 deficiency. Based on these results, we propose that inactivating MMR allows cells to tolerate replication errors and avoid the lethality of futile repair attempts. These findings provide new insights into the genomic plasticity of eukaryotes and enhance our understanding of the mechanisms driving adaptive evolution in response to essential gene insufficiency.

## Data Availability

The original data presented in the study are openly available in SRA database at https://www.ncbi.nlm.nih.gov/sra/PRJNA1372412 (accessed on 19 January 2026).

## Supplementary Materials

The following supporting information can be downloaded at https://www.mdpi.com/article/10.3390/cimb48020158/s1. References [26,53,54] are cited in the Supplementary Materials.
