# Supplementary material for: Genomic Instability and Adaptive Evolution Induced by RFA Insufficiency in Saccharomyces cerevisiae
Source: Curr Issues Mol Biol. 2026 Jan 30;48(2):158. doi: 10.3390/cimb48020158 (PMC12939431; doi:10.3390/cimb48020158)
Supplement: Supplementary file 1 [file cimb-48-00158-s001.zip › Supplemental information.pdf]

Supplemental Tables:

Table S1. Primers used in this study.

Table S2. Strain constructions and strain list

Table S3. Annotations of SNVs variant sites for some L*RFA2*-2 and 5L*RFA2*-2 strains

Supplemental References

1. Sui Y, Qi L, Wu JK, Wen XP, Tang XX et al. Genome-wide mapping of spontaneous genetic alterations in diploid yeast cells. *Proc Natl Acad Sci U S A* **2020**, *117*, 28191-28200.
2. Gueldener U, Heinisch J, Koehler GJ, Voss D, Hegemann JH. A second set of marker cassettes for Cre-mediated multiple gene knockouts in budding yeast. *Nucleic Acids Res* **2002**, *30*, e23. https://doi.org/10.1093/nar/30.6.e23
3. Meng XZ, Wei L, Peng XP, Zhao XL. Sumoylation of the DNA polymerase ε by the Smc5/6 complex contributes to DNA replication. *PLoS Genet* **2019**, *1*, e1008426. https://doi.org/10.1371/journal.pgen.1008426

Table S1 Primers used in this study.

Primers	Sequences（5'-3'）	Purpose
dUNG1-s	ATTCATCCATAATGATATATATTATCAGAAGCTGTACACAAGCTGCAGGTCGACAACCC	Amplification of deletion cassette to delete UNG1 gene
dUNG1-a	TGCTTTTAAAGAGTTTGGAAATCGAGACCTGCATATGCAATAGTGGATCTGATATCACC	
dEXO1-s	CAGGTATATCTATATGCTCTCATAGAATTATATTTGATATTGCTGCAGGTCGACAACCC	Amplification of deletion cassette to delete EXO1 gene
dEXO1-a	AGATTTTCATTTGAAAAATATACCTCCGATATGAAACGTGCAGTGGATCTGATATCACC	
dMLH1-s	TACGATAGTGATAGTAAATGGAAGGTAAAAATAACATAGACGCTGCAGGTCGACAACCC	Amplification of deletion cassette to delete MLH1 gene
dMLH1-a	ACAATCACACTCAGGAAATAAACAAAAAACTTTGGTATTACAGTGGATCTGATATCACC	
dMLH3-s	TACATAAACCAGCGAGGCTTTCAAGGAAGAATGAACGTGAAGCTGCAGGTCGACAACCC	Amplification of deletion cassette to delete MLH3 gene
dMLH3-a	ATATCCGCGCAATTTAAAATGCAGGCGACAAACCTTGTTCCAGTGGATCTGATATCACC	
dMSH2-s	ACACTCTACTCCAATATCAACTGTAAAAAATCTCTTTATCTGCTGCAGGTCGACAACCC	Amplification of deletion cassette to delete MSH2 gene
dMSH2-a	GTACTATTTGTATCTATATATTATCTATCGATTCTCACTTAAGTGGATCTGATATCACC	
dMSH3-s	TGAGAGCCAAAAGCAGTGCAAATAGATTTATTTTGTTGAATGCTGCAGGTCGACAACCC	Amplification of deletion cassette to delete MSH3 gene
dMSH3-a	ATAATATCATTTATTGTCTGATAATGCTGCATTTAGAACATAGTGGATCTGATATCACC	
dMSH6-s	AATTGGAGCAACTAGTTAATTTTGACAAAGCCAATTTGAACGCTGCAGGTCGACAACCC	Amplification of deletion cassette to delete MSH6 gene

dMSH6-a	TTCTGAATCCTTTTTCAACGACCAAAACTTTAAAAAAAATAAGTGGATCTGATATCACC	
dPMS1-s	CAGAAAAAAGGTGTAAGCAAAAGGAACAGAGGTATATCCCGGCATCAGAGCAGATTGTA	Amplification of deletion cassette to delete PMS1 gene
dPMS1-a	ACTCCCTGTATATAATGTATTTGTTAATTATATAATGAATGAGTCAGTGAGCGAGGAAG	
dRFA1p-s	AGGCGAAACCAGCAAGAAGACCAGATTATACTTACAAGAGCAGTATAGCGACCAGCAT	Amplification of deletion cassette to delete RFA1 promotor
dRFA1p-a	AAGATGCTATGAAAATCGCCCCTCGAAAGTTGAACACTGCTAGCGTAATCTGGAACGTC	
dRFA2p-s	TCCTTTATAATAAGCAATATTATATCAACTTAAGGTGGAACTGGATGGCGGCGTTAGTA	Amplification of deletion cassette to delete RFA2 promotor
dRFA2p-a	CAAATCCTCCAATTCCTAGCACTAATACATACTTGCCATGCACTGAGCAGCGTAATCTG	
dRFA3p-s	CCATTCTTGTAGAACATCGTCTACCAGTAACACAAGTAAAACAGTATAGCGACCAGCAT	Amplification of deletion cassette to delete RFA3 promotor
dRFA3p-a	TTGGAGATTTCTGTGGGGTCAACTCTTGGTGTTTCGCTGGCAGCGTAATCTGGAACGTC	
up-3ha-s	ACAAACCTGAGATTGCTTTCA	Amplification of deletion cassette to delete KanMX6-Gal1
up-3ha-a	GGTAAAAGATGTTAATTAAAGACATATGGAGACGTTATTGCATGCA	
down-3ha-s	ATGTCTTTAATTAACATCTTTTACCCAT	Amplification of deletion cassette to delete KanMX6-Gal1
down-3ha-a	TTAGTTTCCGACTCCCCACTA	
vURA3-a	GACGAAGTTGTAAGTGGTGGAT	Verification of insertion of URA3
vUNG1-s	TGGTTGTTCTGTGCTTAGGC	Verification of correct insertion of URA3 at UNG1 location
vEXO1-s	GGTGGAACTGAGGTTGACTACT	Verification of correct insertion of URA3 at EXO1 location
vMLH1-s	CAGTCGGTTGGAAGTGAAGATT	Verification of correct insertion of URA3 at MLH1 location

vMLH3-s	CGTTACTATAACTGCCGATGCT	Verification of correct insertion of URA3 at MLH3 location
vMSH2-s	CTTCTTCTGACGAGCCTCCA	Verification of correct insertion of URA3 at MSH2 location
vMSH3-s	ATCTACGGACGACCTGTCTAAA	Verification of correct insertion of URA3 at MSH3 location
vMSH6-s	GGGAGTTTCAAGGCGGTTTAG	Verification of correct insertion of URA3 at MSH6 location
vPMS1-s	GTTTGTGGGCGTACTGTCAG	Verification of correct insertion of URA3 at PMS1 location
vUP-RFA1-s	CAGAGGTATCCAAGAAGTTGACAT	Verification of correct insertion of KanMX6 at RFA1 promotor location
vUP-RFA2-s	GGCTGTATTAACTGCTCCTATT	Verification of correct insertion of KanMX6 at RFA2 promotor location
vUP-RFA3-s	GACGGCTTCTACTATCATCATTACA	Verification of correct insertion of KanMX6 at RFA3 promotor location
vRFA-a	ATGGTTGATAGGCTGTATTGGA	Verification of correct insertion of KanMX6 at RFA promotor location
IN-UNG1-s	TCGAAGAACTTGAGCAGCAATT	To verify the existence of the UNG1 gene
IN-UNG1-a	CCAGGAACAACACTCCAGTCT	
IN-EXO1-s	ATCCGAAGATTCTGACCTCCTC	To verify the existence of the EXO1 gene
IN-EXO1-a	TCCTACGCTTAATTGCCATTGT	
IN-MLH1-s	GATACGCCATTCATTCCAAGGA	To verify the existence of the MLH1 gene
IN-MLH1-a	GGTTCACTCAGTTGTCGCTTT	
IN-MLH3-s	TTGTCCTTGGAGCCGAACA	To verify the existence of the MLH3 gene
IN-MLH3-a	GAGCGAATGGTCTTAACGATCA	

IN-MSH2-s	GGGTGCGTCGTTACATTATTGA	To verify the existence of the MSH2 gene
IN-MSH2-a	GGCTGTCGTCCTCCAAGAA	
IN-MSH3-s	TGACGCATAAGCAAGAACAAGA	To verify the existence of the MSH3 gene
IN-MSH3-a	GCTCTACCACGGCAACCTT	
IN-MSH6-s	GGTCCAGAAGCCTACTCCAG	To verify the existence of the MSH6 gene
IN-MSH6-a	TGAGCATCTCGTTCATCCACTA	
IN-PMS1-s	CGCAGAGATTGAGCCAGTTG	To verify the existence of the PMS1 gene
IN-PMS1-a	TCGTAACCCACCGTCTTCTTT	

Table S2. Strain constructions and strain list

**Name**	**Background**	***Genotype***	**Construction**
WYspo11	W303 x YJM789	*MAT***a***/α leu2-3,112/LEU his3-11,15/HIS ura3-1/ura3 ade2- 1/ade2-1 trp1- 1/TRP can1-100/CAN1 RAD5/RAD5 GAL2/gal2 ho::hisG IV1510386/IV1510386::SUP4-o spo11::loxP-KanMX6-loxP/spo11::loxP-KanMX6-loxP*	[1]
Wspo11	W303	*MAT***a** *leu2-3,112 his3-11,15 ura3-1 ade2-1 trp1-1 can1-100 RAD5 spo11::loxP-KanMX6-loxP*	[1]
Yspo11	YJM789	*MATα ade2-1 ura3 gal2 ho::hisG IV1510386::SUP4-o spo11::loxP-KanMX6-loxP*	[1]
Wspo11-loxP	W303	*MAT***a** *leu2-3,112 his3-11,15 ura3-1 ade2-1 trp1-1 can1-100 RAD5 spo11::loxP*	Transformed Wspo11 with the Cre-expressing plasmid pSH65 [2] to delete *KanMX6.* Obtained a strain that subsequently lost pSH65.

Yspo11-loxP	YJM789	*MATα ade2-1 ura3 gal2 ho::hisG IV1510386::SUP4-o spo11::loxP*	Deleted *KanMX6* as described for Wspo11-loxP
W*p*GAL1-*RFA1*	W303	*MAT***a** *leu2-3,112 his3-11,15 ura3-1 ade2-1 trp1-1 can1-100 RAD5 spo11::loxP rfa1(ATG):: KanMX6-GAL1-3HA*	The KanMX6-GAL1-3HA cassette primers were amplified with primers containing the homologous terminal of *RFA1* and transformed into Wspo11-loxP to replace the *RFA1* promoter
Y*p*GAL1-*RFA1*	YJM789	*MATα ade2-1 ura3 gal2 ho::hisG IV1510386::SUP4-o spo11::loxP rfa1(ATG):: KanMX6-GAL1-3HA*	replace the *RFA1* promoter as described for W*p*GAL1-*RFA1*
WY*p*GAL1-*RFA1*	W303 x YJM789	*MAT***a***/α leu2-3,112/LEU2 his3-11,15/HIS3 ura3-1/ura3 ade2-1/ade2-1 trp1-1/TRP1 can1-100/CAN1 GAL2/gal2 ho::hisG/ho IV1510386/IV1510386::SUP4-o spo11::loxP /spo11::loxP rfa1(ATG):: KanMX6-GAL1-3HA / rfa1(ATG):: KanMX6-GAL1-3HA*	Mating of W*p*GAL1-*RFA1* with Y*p*GAL1-*RFA1*. For sub-cultured WY*p*GAL1-*RFA1* strains .
W*p*GAL1-*RFA2*	W303	*MAT***a** *leu2-3,112 his3-11,15 ura3-1 ade2-1 trp1-1 can1-100 RAD5 spo11::loxP rfa2(ATG):: KanMX6-GAL1-3HA*	The KanMX6-GAL1-3HA cassette primers were amplified with primers containing the homologous terminal of *RFA2* and transformed into Wspo11-loxP to replace the *RFA2* promoter
Y*p*GAL1-*RFA2*	YJM789	*MATα ade2-1 ura3 gal2 ho::hisG IV1510386::SUP4-o spo11::loxP rfa2(ATG):: KanMX6-GAL1-3HA*	replace the *RFA2* promoter as described for W*p*GAL1-*RFA2*

WY*p*GAL1-*RFA2*	W303 x YJM789	*MAT***a***/α leu2-3,112/LEU2 his3-11,15/HIS3 ura3-1/ura3 ade2-1/ade2-1 trp1-1/TRP1 can1-100/CAN1 GAL2/gal2 ho::hisG/ho IV1510386/IV1510386::SUP4-o spo11::loxP /spo11::loxP rfa2(ATG):: KanMX6-GAL1-3HA / rfa2(ATG):: KanMX6-GAL1-3HA*	Mating of W*p*GAL1-*RFA2* with Y*p*GAL1-*RFA2*. For sub-cultured WY*p*GAL1-*RFA2* strains.<br>a. like WY123#3-3 indicates individual isolates sub-cultured (single cell to a colony) 3 times at 30 °C on LG. The last number indicates the individual isolates in Dataset S1.<br>b. WY123#5-1 indicates isolates sub-cultured 5 times at 30 °C on LG (see Dataset S1).
W*RFA2−ha*	W303	*MAT***a** *leu2-3,112 his3-11,15 ura3-1 ade2-1 trp1-1 can1-100 RAD5 spo11::loxP*	Two pairs of primers, up-3ha-s, up-3ha-a and down-3ha-s, down-3ha-a were used for amplification respectively. Bridge PCR was adopted to obtain cassette, and W*p*GAL1-*RFA2* was transferred to replace KanMX6-GAL1 as the ATG promoter, while the 3× HA tag was retained.
W*p*GAL1-*RFA3*	W303	*MAT***a** *leu2-3,112 his3-11,15 ura3-1 ade2-1 trp1-1 can1-100 RAD5 spo11::loxP rfa3(ATG):: KanMX6-GAL1-3HA*	The KanMX6-GAL1-3HA cassette primers were amplified with primers containing the homologous terminal of *RFA3* and transformed into Wspo11-loxP to replace the *RFA3* promoter

Y*p*GAL1-*RFA3*	YJM789	*MATα ade2-1 ura3 gal2 ho::hisG IV1510386::SUP4-o spo11::loxP rfa3(ATG):: KanMX6-GAL1-3HA*	replace the *RFA3* promoter as described for W*p*GAL1-*RFA3*
WY*p*GAL1-*RFA3*	W303 x YJM789	*MAT***a***/α leu2-3,112/LEU2 his3-11,15/HIS3 ura3-1/ura3 ade2-1/ade2-1 trp1-1/TRP1 can1-100/CAN1 GAL2/gal2 ho::hisG/ho IV1510386/IV1510386::SUP4-o spo11::loxP /spo11::loxP rfa3(ATG):: KanMX6-GAL1-3HA / rfa3(ATG):: KanMX6-GAL1-3HA*	Mating of W*p*GAL1-*RFA3* with Y*p*GAL1-*RFA3*. For sub-cultured WY*p*GAL1-*RFA3* strains. a. like WY124#5-21 indicates individual isolates sub-cultured (single cell to a colony) 5 times at 30 °C on LG. The last number indicates the individual isolates in Dataset S2.<br>b. WY124#6-41 indicates isolates sub-cultured 6 times at 30 °C on LG (see Dataset S2).
X8068-6C	W303	*MAT***a** *can1-100 ura3-1 his3-11,15 leu2-3,112 trp1-1 RAD52-YFP*	[3]
X8068-6Crfa2	W303	*MAT***a** *can1-100 ura3-1 his3-11,15 leu2-3,112 trp1-1 RAD52-YFP rfa2(ATG):: KanMX6-GAL1-3HA*	The construction method is similar to W*p*GAL1-*RFA2*.
W*p*GAL1-*RFA2* d*EXO1*	W303	*MAT***a** *leu2-3,112 his3-11,15 ura3-1 ade2-1 trp1-1 can1-100 RAD5 spo11::loxP rfa2(ATG):: KanMX6-GAL1-3HA exo1::URA3*	*Exo1* deletion generated by transformation of W*p*GAL1-*RFA2* with PCR fragment obtained by amplifying pUG72 plasmid DNA with primers dEXO1-s and dEXO1-a
W*p*GAL1-*RFA2* d*MLH1*	W303	*MAT***a** *leu2-3,112 his3-11,15 ura3-1 ade2-1 trp1-1 can1-100 RAD5 spo11::loxP rfa2(ATG):: KanMX6-GAL1-3HA mlh1::URA3*	*MLH1* deletion generated by transformation of W*p*GAL1-*RFA2* with PCR fragment obtained by amplifying pUG72 plasmid DNA with primers dMLH1-s and dMLH1-a

W*p*GAL1-*RFA2* d*MLH3*	W303	*MAT***a** *leu2-3,112 his3-11,15 ura3-1 ade2-1 trp1-1 can1-100 RAD5 spo11::loxP rfa2(ATG):: KanMX6-GAL1-3HA mlh3::URA3*	*MLH3* deletion generated by transformation of W*p*GAL1-*RFA2* with PCR fragment obtained by amplifying pUG72 plasmid DNA with primers dMLH3-s and dMLH3-a
W*p*GAL1-*RFA2* d*MSH2*	W303	*MAT***a** *leu2-3,112 his3-11,15 ura3-1 ade2-1 trp1-1 can1-100 RAD5 spo11::loxP rfa2(ATG):: KanMX6-GAL1-3HA msh2::URA3*	*MSH2* deletion generated by transformation of W*p*GAL1-*RFA2* with PCR fragment obtained by amplifying pUG72 plasmid DNA with primers dMSH2-s and dMSH2-a
W*p*GAL1-*RFA2* d*MSH3*	W303	*MAT***a** *leu2-3,112 his3-11,15 ura3-1 ade2-1 trp1-1 can1-100 RAD5 spo11::loxP rfa2(ATG):: KanMX6-GAL1-3HA msh3::URA3*	*MSH3* deletion generated by transformation of W*p*GAL1-*RFA2* with PCR fragment obtained by amplifying pUG72 plasmid DNA with primers dMSH3-s and dMSH3-a
W*p*GAL1-*RFA2* d*MSH6*	W303	*MAT***a** *leu2-3,112 his3-11,15 ura3-1 ade2-1 trp1-1 can1-100 RAD5 spo11::loxP rfa2(ATG):: KanMX6-GAL1-3HA msh6::URA3*	*MSH6* deletion generated by transformation of W*p*GAL1-*RFA2* with PCR fragment obtained by amplifying pUG72 plasmid DNA with primers dMSH6-s and dMSH6-a
W*p*GAL1-*RFA2* d*PMS1*	W303	*MAT***a** *leu2-3,112 his3-11,15 ura3-1 ade2-1 trp1-1 can1-100 RAD5 spo11::loxP rfa2(ATG):: KanMX6-GAL1-3HA pms1::URA3*	*PMS1* deletion generated by transformation of W*p*GAL1-*RFA2* with PCR fragment obtained by amplifying pUG72 plasmid DNA with primers dPMS1-s and dPMS1-a

Wd*UNG1*	W303	*MAT***a** *leu2-3,112 his3-11,15 ura3-1 ade2-1 trp1-1 can1-100 RAD5 spo11::loxP ung1::URA3*	*UNG1* deletion generated by transformation of Wspo11-loxP with PCR fragment obtained by amplifying pUG72 plasmid DNA with primers dUNG1-s and dUNG1-a.
Yd*UNG1*	YJM789	*MATα ade2-1 ura3 gal2 ho::hisG IV1510386::SUP4-o spo11::loxP ung1::URA3*	*UNG1* deletion generated by transformation of Yspo11-loxP with PCR fragment obtained by amplifying pUG72 plasmid DNA with primers dUNG1-s and dUNG1-a.
WYd*UNG1*	W303 x YJM789	*MAT***a***/α leu2-3,112/LEU2 his3-11,15/HIS3 ura3-1/ura3 ade2-1/ade2-1 trp1-1/TRP1 can1-100/CAN1 GAL2/gal2 ho::hisG/ho IV1510386/IV1510386::SUP4-o spo11::loxP /spo11::loxP ung1::URA3 / ung1::URA3*	Mating of Wd*UNG1* with Yd*UNG1*. For sub-cultured WYd*UNG1* strains. For example, WY105#5-1 indicates that a single isolate was subcultured 5 times at 30°C after being transferred into the pSR440 plasmid (from a single cell to a colony). The last number indicates the individual isolates in Dataset S3.
W*p*GAL1-*RFA1* d*UNG1*	W303	*MAT***a** *leu2-3,112 his3-11,15 ura3-1 ade2-1 trp1-1 can1-100 RAD5 spo11::loxP ung1::URA3 rfa1(ATG):: KanMX6-GAL1-3HA*	The KanMX6-GAL1-3HA cassette primers were amplified with primers containing the homologous terminal of *RFA1* and transformed into Wd*UNG1* to replace the *RFA1* promoter.
Y*p*GAL1-*RFA1* d*UNG1*	YJM789	*MATα ade2-1 ura3 gal2 ho::hisG IV1510386::SUP4-o spo11::loxP ung1::URA3 rfa1(ATG):: KanMX6-GAL1-3HA*	replace the *RFA1* promoter as described for W*p*GAL1-*RFA1* d*UNG1*

WY*p*GAL1-*RFA1* d*UNG1*	W303 x YJM789	*MAT***a***/α leu2-3,112/LEU2 his3-11,15/HIS3 ura3-1/ura3 ade2-1/ade2-1 trp1-1/TRP1 can1-100/CAN1 GAL2/gal2 ho::hisG/ho IV1510386/IV1510386::SUP4-o spo11::loxP /spo11::loxP ung1::URA3 / ung1::URA3 rfa1(ATG):: KanMX6-GAL1-3HA / rfa1(ATG):: KanMX6-GAL1-3HA*	Mating of W*p*GAL1-*RFA1* d*UNG1* with Y*p*GAL1-*RFA1* d*UNG1*. For sub-cultured WY*p*GAL1-*RFA1* d*UNG1* strains. For example, WY105122#5-1 indicates that a single isolate was subcultured 5 times at 30°C on LG after being transferred into the pSR440 plasmid (from a single cell to a colony). The last number indicates the individual isolates in Dataset S3.
W*p*GAL1-*RFA2* d*UNG1*	W303	*MAT***a** *leu2-3,112 his3-11,15 ura3-1 ade2-1 trp1-1 can1-100 RAD5 spo11::loxP ung1::URA3 rfa2(ATG):: KanMX6-GAL1-3HA*	The KanMX6-GAL1-3HA cassette primers were amplified with primers containing the homologous terminal of *RFA2* and transformed into Wd*UNG1* to replace the *RFA2* promoter.
Y*p*GAL1-*RFA2* d*UNG1*	YJM789	*MATα ade2-1 ura3 gal2 ho::hisG IV1510386::SUP4-o spo11::loxP ung1::URA3 rfa2(ATG):: KanMX6-GAL1-3HA*	replace the *RFA2* promoter as described for W*p*GAL1-*RFA2* d*UNG1*

WY*p*GAL1-*RFA2* d*UNG1*	W303 x YJM789	*MAT***a***/α leu2-3,112/LEU2 his3-11,15/HIS3 ura3-1/ura3 ade2-1/ade2-1 trp1-1/TRP1 can1-100/CAN1 GAL2/gal2 ho::hisG/ho IV1510386/IV1510386::SUP4-o spo11::loxP /spo11::loxP ung1::URA3 / ung1::URA3 rfa2(ATG):: KanMX6-GAL1-3HA / rfa2(ATG):: KanMX6-GAL1-3HA*	Mating of W*p*GAL1-*RFA2* d*UNG1* with Y*p*GAL1-*RFA2* d*UNG1*. For sub-cultured WY*p*GAL1-*RFA2* d*UNG1* strains. For example, WY105123#1-1 indicates that a single isolate was subcultured 1 time at 30°C on LG after being transferred into the pSR440 plasmid (from a single cell to a colony). The last number indicates the individual isolates in Dataset S3.
W*p*GAL1-*RFA3* d*UNG1*	W303	*MAT***a** *leu2-3,112 his3-11,15 ura3-1 ade2-1 trp1-1 can1-100 RAD5 spo11::loxP ung1::URA3 rfa3(ATG):: KanMX6-GAL1-3HA*	The KanMX6-GAL1-3HA cassette primers were amplified with primers containing the homologous terminal of *RFA3* and transformed into Wd*UNG1* to replace the *RFA3* promoter.
Y*p*GAL1-*RFA3* d*UNG1*	YJM789	*MATα ade2-1 ura3 gal2 ho::hisG IV1510386::SUP4-o spo11::loxP ung1::URA3 rfa3(ATG):: KanMX6-GAL1-3HA*	replace the *RFA3* promoter as described for W*p*GAL1-*RFA3* d*UNG1*

WY*p*GAL1-*RFA3* d*UNG1*	W303 x YJM789	*MAT***a***/α leu2-3,112/LEU2 his3-11,15/HIS3 ura3-1/ura3 ade2-1/ade2-1 trp1-1/TRP1 can1-100/CAN1 GAL2/gal2 ho::hisG/ho IV1510386/IV1510386::SUP4-o spo11::loxP /spo11::loxP ung1::URA3 / ung1::URA3 rfa3(ATG):: KanMX6-GAL1-3HA / rfa3(ATG):: KanMX6-GAL1-3HA*	Mating of W*p*GAL1-*RFA3* d*UNG1* with Y*p*GAL1-*RFA3* d*UNG1*. For sub-cultured WY*p*GAL1-*RFA3* d*UNG1* strains. For example, WY105124#1-1 indicates that a single isolate was subcultured 1 time at 30°C on LG after being transferred into the pSR440 plasmid (from a single cell to a colony). The last number indicates the individual isolates in Dataset S3.

Table S3. Annotations of SNVs variant sites for some L*RFA2*-2 and 5L*RFA2*-2 strains

Strains	Chromosome	Position	SNP_before	SNP_after	Gene
WY123#5-1	chrIV	641811	A	T	*MSH6*
WY123#5-3	chrIII	279451	CT	C	*MSH3*
WY123#5-3	chrXII	212656	C	CT	*MLH2*
WY123#5-5	chrXIII	597173	ACAAAGTTTTGAGAGGT…	A	*MLH1*
LWY123#5-1	chrXIV	434356	G	T	*POL1*
LWY123#5-4	chrXIII	597173	ACAAAGTTTTGAGAGGTG…	A	*MLH1*
LWY123#5-6	chrIV	641392	G	A	*MSH6*
LWY123#5-8	chrIII	277967	G	A	*MSH3*
LWY123#5-8	chrXIV	473981	TA	T	*PMS1*
LWY123#5-14	chrXII	212569	C	G	*MLH2*

Note: Only a subset of MMR-related mutations is listed, and all of these mutations have a HIGH or MODERATE impact on the gene. WY123 stands for L*RFA2*; LWY123 stands for 5L*RFA2*.
